# Value of anti-hepatitis B virus in serum tested by enzyme linked immunosorbent assay 3 in diagnosis of hepatitis B: A protocol for systemic review and meta analysis

**DOI:** 10.1097/MD.0000000000023961

**Published:** 2021-01-22

**Authors:** Shen Fei-fei, Ren Xiao-dan, Liu Hai-qiang, Zhang He-wei

**Affiliations:** a Department of Medical Laboratory Technology, Luoyang Polytechnic; b Department of Traditional Chinese Medicine, The Second Affiliated Hospital of Army Medical University; c Department of Medical Imaging Technology, Luoyang Polytechnic; d The college of Food and Drugs, Luoyang Polytechnic.

**Keywords:** enzyme linked immunosorbent assay, hepatitis B virus, systematic review

## Abstract

**Introduction:**

This protocol is for a meta analysis that aims to systematically review the diagnostic value of anti-hepatitis B virus in serum tested by the enzyme linked immunosorbent assay in patients with hepatitis B.

**Methods and analysis:**

The following electronic databases will be searched from inception to Mar 2021: PubMed, Web of Science, ScienceDirect, EMBASE, MEDLINE, Springer, China National Knowledge Infrastructure, Chinese Biomedical Literature Database, VIP Chinese Science and Technology Periodical Database, and Wanfang Database. All study about enzyme linked immunosorbent assay reagents have been published at home and abroad to diagnose hepatitis B virus will be included. MetaDisc 1.4 soft will used to calculate pooled effect size in sensitivity, specifi city, likelihood ratio, diagnostic odds ratio and summary receiver operating characteristic curve, and area under the curve as well.

**Ethics and dissemination:**

Formal ethical approval is not required, as the data are not individualized. The findings of this systematic review will be disseminated in a peer-reviewed publication and/or presented at relevant conferences.

**Registration number:**

INPLASY2020100051.

## 1 Introduction

Hepatitis B virus (HBV) infection is a global public health problem.^[1]^ There are about 400 million chronically infected people, nearly One-third of people have been infected with HBV, especially in developing countries.^[2]^ China is a high-risk area of viral hepatitis, especially hepatitis B virus Inflammation, not only the population infection rate is high, but also easy to turn into chronic hepatitis, some cases can evolve into liver cirrhosis or even primary liver cancer.^[3–6]^ Therefore, early diagnosis and early treatment of hepatitis B patients is particularly important want. The enzyme linked immunosorbent assay (ELISA) method is simple to operate, the results are easy to judge, the price is low, no special equipment and high standard experimental conditions are needed, and it is easy to popularize and apply.^[7,8]^ In order to evaluate the value of ELISA reagents in the diagnosis of HBV, this study will comprehensively collect relevant literature for meta-analysis, in order to provide more reliable evidence for the clinic.

## 2 Methods and analysis

### 2.1 Study registration

The protocol for this systematic review was registered with INPLASY (registration number: INPLASY2020100051). This protocol report is based on the Preferred Reporting Items for Systematic Reviews and Meta-Analyses Protocols (PRISMA-P) guidelines.^[9]^ The review will be conducted in accordance with the PRISMA guidelines.

### 2.2 Inclusion criteria for study selection

#### 2.2.1 Type of study

ELISA reagents have been published at home and abroad to diagnose HBV will be eligible for inclusion, without restrictions on publication status.

#### 2.2.2 Type of participant

Participants aged 18 years or older with HBV patients will be included, regardless of their sex, race, education level, or economic status.

#### 2.2.3 Type of diagnosis

Anti-HBV in Serum Tested by ELISA3

#### 2.2.4 Type of outcome measure

Pooled effect size in sensitivity, specifi city, likelihood ratio, diagnostic odds ratio and summary receiver operating characteristic curve, and area under the curve as well.^[10]^

### 2.3 Search methods for identification of studies

#### 2.3.1 Data sources

The following electronic databases will be searched from inception to Mar 2021: PubMed, Web of Science, ScienceDirect, EMBASE, MEDLINE, Springer, China National Knowledge Infrastructure, Chinese Biomedical Literature Database, VIP Chinese Science and Technology Periodical Database, and Wanfang Database. All study about ELISA reagents have been published at home and abroad to diagnose HBV will be included.

#### 2.3.2 Searching other resources

We will scan the reference lists of retrieved studies to identify other eligible studies. Relevant conference proceedings will also be searched.

#### 2.3.3 Search strategy

The search strategy for PubMed is shown in Table 1. The following keywords will be used: hepatitis B; ELISA; the third-generation ELISA; Anti-HBV; diagnosis; sensitivity; specificity; PCR; HBV-RNA. The equivalent search keywords will be used in the Chinese databases.

**Table 1:** Search strategy for the PubMed database.

Number	Search items
1	Hepatitis B. Mesh.
2	Hepatitis B. ti, ab
3	1 or 2
4	ELISA. Mesh.
5	ELISA. ti, ab
6	the third-generation ELISA. ti, ab
7	4 or 5,6
8	Anti-HBV. Mesh.
9	Anti-HBV ti, ab
10	8 or 9
11	Diagnosis. Mesh.
12	Diagnosis. ti, ab
13	11 or 12
14	Sensitivity. Mesh.
15	Sensitivity.. ti, ab
16	14 or 15
17	Specificity. Mesh.
18	Specificity.. ti, ab
19	17 or 18
20	PCR. Mesh.
21	PCR.. ti, ab
22	20 or 21
23	HBV-RNA. Mesh.
24	HBV-RNA.. ti, ab
25	23 or 24
26	3 and 7 and 10 and (13 or 16 or 19) and (22 or 25)

### 2.4 Data collection and analysis

#### 2.4.1 Selection of studies

**Figure 1:**
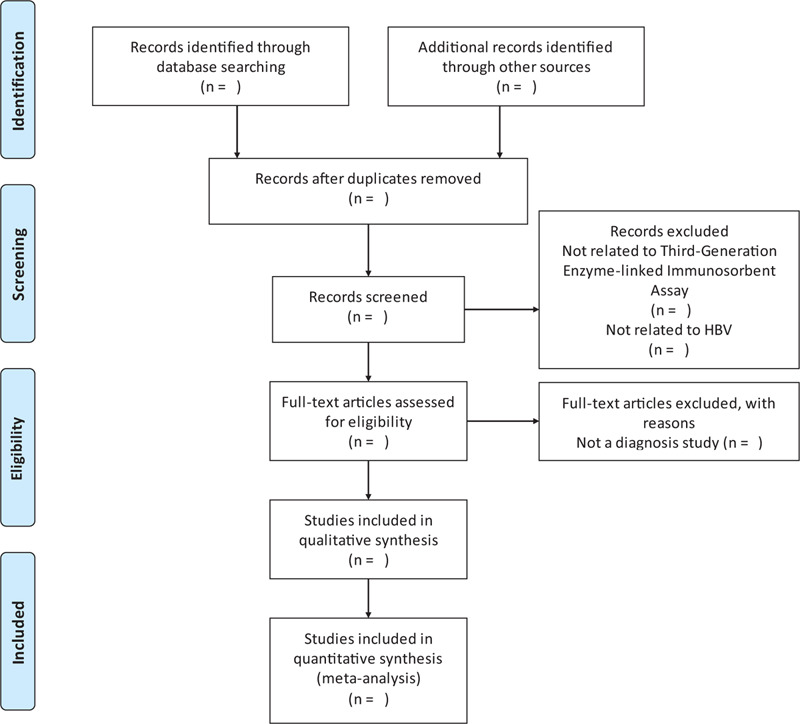
The PRISMA flow chart of the selection process.

Two reviewers will independently review and screen the titles and abstracts of all retrieved studies to identify eligible trials and eliminate duplicated or irrelevant studies in accordance with the inclusion and exclusion criteria; the full text of all potentially eligible studies will then be obtained. Any disagreements will be resolved by discussion with a third reviewer. The study selection process is shown in a PRISMA flow diagram (Fig. 1).

#### 2.4.2 Data extraction and management

The following data will be extracted from the selected studies by 2 independent reviewers using a standard data extraction sheet: year of publication, country, general information, participant characteristics, sample size, gold standard, true positive value, false positive value, true negative value, and false negative value. Any disagreements will be resolved by discussion with a third reviewer. For publications with insufficient data, we will attempt to obtain the missing data from the authors. All data will be transferred into Meta-Disc 1.4 for analysis and synthesis.

#### 2.4.3 Assessment of risk of bias in included studies

For all included studies, 2 reviewers will independently evaluate the risk of bias using the Cochrane Collaboration's tool assessment method. The risks of bias will be categorized into 3 levels (low risk, high risk, and unclear) in accordance with the following domains: sequence generation, allocation concealment, blinding of outcome assessors and participants, incomplete outcome data, selective outcome reporting, and other sources of bias. We will attempt to clarify unclear or insufficient items by contacting the corresponding author for more details. Any discrepancies will be resolved by discussion with a third reviewer.

#### 2.4.4 Unit of analysis

The analytical unit will be the individual participant.

#### 2.4.5 Management of missing data

The corresponding authors of the included studies will be contacted by reviewers to retrieve any missing or insufficient data of the primary results. If missing data is not available, an intent-to-treat analysis will be performed, and a sensitivity analysis will be performed to determine whether the results are inconsistent.

#### 2.4.6 Assessment of heterogeneity

We will use the standard χ^2^ test to detect statistical heterogeneity, with the I^2^ test to quantify inconsistency. When the *P* value exceeds .1, and the *I*^2^ value is less than 50%, studies will be considered homogeneous, and the fixed-effects model will be used. When the *P* value is less than .1, or the *I*^2^ value exceeds 50%, studies will be considered to have significant statistical heterogeneity, and subgroup analysis will be performed to explore the possible cause; if the heterogeneity remains significant, the random-effects model will be used.

#### 2.4.7 Assessment of reporting biases

If more than 10 studies are included, funnel plots will be used to detect potential reporting biases. The Egger test will be used to determine asymmetry of the funnel plots.

#### 2.4.8 Data synthesis

The fixed-effects model will be used for data synthesis if no substantial statistical heterogeneity is detected, while the random-effects model will be used if there is substantial statistical heterogeneity. If there is significant heterogeneity between studies, we will search for possible causes from a clinical and methodological perspective, and provide a descriptive analysis or subgroup analysis.

#### 2.4.9 Subgroup analysis

Subgroup analysis will be performed to explain heterogeneity if possible. Factors such as different types of control interventions and different outcomes will be considered.

#### 2.4.10 Sensitivity analysis

If possible, sensitivity analyses will be conducted to verify the robustness of the review conclusions. The impacts of sample size, study design, methodological quality, and missing data will be evaluated. The analysis will be repeated after the exclusion of studies with low methodological quality.

#### 2.4.11 Grading the quality of evidence

The quality of the evidence will be judged using the Grade of Recommendations Assessment, Development and Evaluation.^[11]^ The following criteria will be assessed: limitations of the study design, inconsistency of results, imprecision, indirectness, and publication bias. The quality of included studies will be classified into 4 levels: high, moderate, low, or very low.

## 3 Ethics and dissemination

Ethics approval will not be needed because the data that will be used are not individual and no privacy will be involved. The results will be disseminated through peer-reviewed publications or conference presentations. The essential protocol amendments will be documented in the full review.

## 4 Discussion

This systematic review will be the first to assess the Value of anti-HBV in serum tested by

ELISA3 in diagnosis of hepatitis B, and its results will address a gap in the literature. The review will be separated into 4 sections: identification, study inclusion, data extraction, and data synthesis. We believe that this review will aid practitioners in the decision-making process for diagnosis of hepatitis B, and will provide important information for patients and health policy makers.

## Author contributions

FFS and XDR contributed equally to this manuscript and joint first authors. FFS and XDR drafted the protocol. The search strategy was developed and will be conducted by FFS. HQL will obtain copies of the studies and select the studies to be included. XDR extract data from the studies. FFS will conduct the analyses. HWZ will act as an arbiter in the study selection stage. All authors have read and approved the final manuscript.

**Data curation:** Liu Hai-qiang.

**Methodology:** Ren Xiao-dan.

**Supervision:** Hewei Zhang.

**Visualization:** Liu Hai-qiang.

**Writing – original draft:** Shen Fei-fei, Ren Xiao-dan.

**Writing – review & editing:** Shen Fei-fei, Hewei Zhang.
